# *Trans*-Synaptic Signaling through the Glutamate Receptor Delta-1 Mediates Inhibitory Synapse Formation in Cortical Pyramidal Neurons

**DOI:** 10.1016/j.neuron.2019.09.027

**Published:** 2019-12-18

**Authors:** Matteo Fossati, Nora Assendorp, Olivier Gemin, Sabrina Colasse, Florent Dingli, Guillaume Arras, Damarys Loew, Cécile Charrier

**Affiliations:** 1 Institut de Biologie de l’Ecole Normale Supérieure (IBENS), Ecole Normale Supérieure, CNRS, INSERM, PSL Research University, 75005 Paris, France; 2 Institut Curie, PSL Research University, Centre de Recherche, Laboratoire de Spectrométrie de Masse Protéomique, 75248 Paris Cedex 05, France

**Keywords:** glutamate receptor delta-1, gephyrin, synaptogenesis, inhibitory synapse, trans-synaptic interaction, synaptic signaling, cerebellin, synapse specificity, adhesion molecule, cortical circuits

## Abstract

Fine orchestration of excitatory and inhibitory synaptic development is required for normal brain function, and alterations may cause neurodevelopmental disorders. Using sparse molecular manipulations in intact brain circuits, we show that the glutamate receptor delta-1 (GluD1), a member of ionotropic glutamate receptors (iGluRs), is a postsynaptic organizer of inhibitory synapses in cortical pyramidal neurons. GluD1 is selectively required for the formation of inhibitory synapses and regulates GABAergic synaptic transmission accordingly. At inhibitory synapses, GluD1 interacts with cerebellin-4, an extracellular scaffolding protein secreted by somatostatin-expressing interneurons, which bridges postsynaptic GluD1 and presynaptic neurexins. When binding to its agonist glycine or D-serine, GluD1 elicits non-ionotropic postsynaptic signaling involving the guanine nucleotide exchange factor ARHGEF12 and the regulatory subunit of protein phosphatase 1 PPP1R12A. Thus, GluD1 defines a *trans*-synaptic interaction regulating postsynaptic signaling pathways for the proper establishment of cortical inhibitory connectivity and challenges the dichotomy between iGluRs and inhibitory synaptic molecules.

## Highlights

• GluD1 mediates the formation and specification of inhibitory synapses in the cortex
• GluD1 forms *trans*-synaptic interactions via Cbln4, a protein secreted by SST^+^ INs
• GluD1 elicits agonist-dependent postsynaptic signaling via ARHGEF12 and PPP1R12A
• GluD1 is a maverick iGluR operating through non-ionotropic mechanisms

## Teaser

Fossati et al. show that, in stark contrast to other iGluRs, the glutamate receptor delta-1 (GluD1) operates at inhibitory, rather than excitatory, synapses in the cortex. GluD1 mediates *trans*-synaptic signaling governing the formation of synapses between specific interneurons and pyramidal neurons.

## Introduction

Synapses constitute the elementary functional units of the brain. They convey excitatory or inhibitory signals that need to be coordinated in space and time for optimal brain function (Mullins et al., 2016, Nelson and Valakh, 2015). Excitatory and inhibitory synapses in the mammalian brain mainly use glutamate and gamma-aminobutyric acid (GABA) as a neurotransmitter, respectively. They are multi-molecular nanomachines composed of almost exclusive sets of proteins (Krueger-Burg et al., 2017, Sheng and Kim, 2011, Tyagarajan and Fritschy, 2014). Yet they share the same basic organization, which ensures the efficacy and fine-tuning of synaptic transmission. The organization of synapses relies on transient, highly regulated interactions between various categories of proteins (neurotransmitter receptors, scaffolding proteins, adhesion proteins, signaling molecules, and cytoskeleton elements) and accommodates a great level of molecular diversity (Choquet and Triller, 2013, Emes and Grant, 2012, Sheng and Hoogenraad, 2007, Tyagarajan and Fritschy, 2014, Ziv and Fisher-Lavie, 2014). The molecular diversity of synapses enables the establishment of complex neuronal networks: it specifies their functional properties and shapes the transfer of information between neurons throughout the brain. Hence, synaptic dysfunctions cause a wide range of neurodevelopmental and psychiatric disorders, such as epilepsy, autisms, or schizophrenia (Bourgeron, 2015, Mullins et al., 2016, Nelson and Valakh, 2015, Ting et al., 2012, Zoghbi and Bear, 2012). *Trans*-synaptic molecular interactions critically contribute to both the development and diversification of synaptic connections. They instruct the formation of synapses following initial contact (McAllister, 2007, Missler et al., 2012), match pre- and post-synaptic neurons (Berns et al., 2018, de Wit and Ghosh, 2016), control the recruitment of neurotransmitter receptors and postsynaptic scaffolding proteins (Aoto et al., 2013, Bemben et al., 2015, Fukata et al., 2006, Lovero et al., 2015, Mondin et al., 2011, Nam and Chen, 2005, Poulopoulos et al., 2009), and regulate synaptic plasticity (Bemben et al., 2015, Jang et al., 2017, Tai et al., 2008, Yuzaki and Aricescu, 2017). Nonetheless, frequent discrepancies between *in vitro* and *in vivo* studies have made the role of some *trans*-synaptic molecular interactions difficult to precisely delineate. Furthermore, the scarcity of information on how *trans*-synaptic signals are transduced in the post-synaptic neuron stymies our understanding of the molecular logic governing the assembly of synaptic connections.

Individual synaptic proteins may operate through a diversity of modalities. Recently, it has emerged that ionotropic glutamate receptors (iGluRs), which are the main excitatory neurotransmitter receptors in the CNS, do not solely operate through ionotropic mechanisms (Dore et al., 2015, Rodríguez-Moreno and Sihra, 2007, Yuzaki and Aricescu, 2017). At least some iGluRs engage in *trans*-synaptic interactions along with conventional cell adhesion molecules (CAMs) (Matsuda et al., 2010, Matsuda et al., 2016, Uemura et al., 2010) or mediate non-ionotropic signaling critical for synaptic development and plasticity (Babiec et al., 2014, Carter and Jahr, 2016, Dore et al., 2015, Grabauskas et al., 2007, Hayashi et al., 1999, Kakegawa et al., 2011, Lerma and Marques, 2013, Nabavi et al., 2013, Saglietti et al., 2007, Stein et al., 2015). Alternative functions of iGluRs are best characterized for the glutamate receptor delta-2 (GluD2), an iGluR of the delta subfamily (comprising GluD1 and GluD2 receptor subunits, encoded by the genes *grid1* and *grid2*) predominantly expressed in the cerebellum (Araki et al., 1993). GluD2 is confined in the postsynaptic membrane of excitatory synapses between parallel fibers (PFs) and Purkinje cells (PCs). It contributes to synaptic adhesion by interacting with presynaptic neurexins containing an insert in the splice site 4 through the extracellular scaffolding proteins cerebellins (Cblns), presynaptically secreted molecules that belong to the C1q family of the classical complement pathway (Südhof, 2017, Uemura et al., 2010, Yuzaki, 2017, Yuzaki, 2018). This *trans*-synaptic interaction controls the specification and maintenance of PF-PC synapses. In addition, activation of GluD2 by its agonist initiates signaling cascades regulating the local accumulation of α-amino-3-hydroxy-5-methyl-4-isoxazolepropionic acid (AMPA) receptors and long-term depression (Kakegawa et al., 2011, Yuzaki and Aricescu, 2017). GluD1 can also form triads with Cblns and neurexins (Yasumura et al., 2012, Yuzaki and Aricescu, 2017). It is widely expressed in the neocortex, hippocampus, striatum, and cerebellum, where its expression is strongly upregulated during the period of synaptogenesis and remains high in adults (Hepp et al., 2015, Konno et al., 2014). GluD1 has been implicated in the formation of excitatory synapses in the cerebellum (Konno et al., 2014) and hippocampus (Tao et al., 2018) and in pruning in the hippocampus and medial prefontal cortex (Gupta et al., 2015). Other studies have suggested a role in the firing of dopaminergic neurons or at inhibitory synapses (Benamer et al., 2018, Ryu et al., 2012, Yasumura et al., 2012). Notwithstanding, GluD1 function remains poorly understood.

In the present study, we have investigated the role of GluD1 in the development of excitatory and inhibitory synapses in the somato-sensory cortex. By depleting GluD1 *in vivo* in a few layer 2/3 cortical pyramidal neurons (CPNs) using sparse *in utero* electroporation (IUE), we demonstrate that GluD1 regulates the formation of inhibitory synapses in dendrites as well as inhibitory synaptic transmission. In contrast, GluD1 is dispensable for the formation and maintenance of excitatory synapses in CNPs. Using an *in vivo* structure/function analysis, we demonstrate that the regulation of inhibitory synapses by GluD1 requires *trans*-synaptic interaction via Cbln4, an extracellular scaffolding protein secreted by somatostatin-expressing (SST^+^) interneurons (INs) (Favuzzi et al., 2019), activation of the receptor by its endogenous agonists glycine and D-Serine, and post-synaptic signaling via the intracellular C-terminal tail of the receptor. Using mass spectrometry, we characterize GluD1 interactome in developing synapses. We show that GluD1 serves as a hub for molecules implicated in inhibitory synaptogenesis, and we identify two major partners of GluD1, the signaling molecules rho guanine nucleotide exchange factor 12 (ARHGEF12) and protein phosphatase 1 regulatory subunit 12A (PPP1R12A), as critical regulators of inhibitory synapse formation in CPNs. Together, our results define a *trans*-synaptic signaling pathway centered on an atypical iGluR for the formation and specification of cortical inhibitory circuits.

## Results

### GluD1 Is Selectively Required for the Formation of Inhibitory Synapses

**Figure 1:**
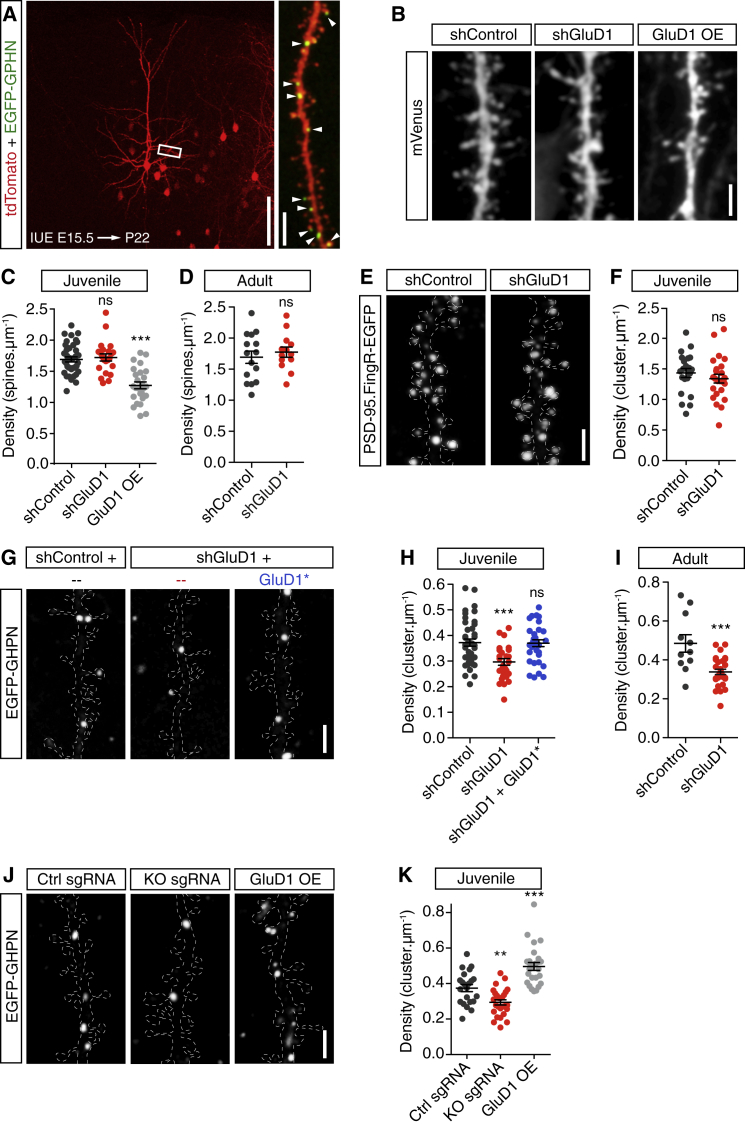
Selective Control of Inhibitory Synapse Density by GluD1 in CPNs (A) Sparse labeling of layer 2/3 CPNs after *in utero* electroporation (IUE) with soluble tdTomato (red) and EGFP-gephyrin (EGFP-GPHN, green). Arrowheads in the enlarged area highlight inhibitory synapses in oblique apical dendrites. E15.5, embryonic day 15.5; P22: postnatal day 22. Scale bars: 100 μm (left) and 5 μm (right). (B) Segments of dendrites expressing shControl or shGluD1 or overexpressing (OE) GluD1 along with mVenus to visualize dendritic spines in juvenile mice. Scale bar: 2 μm. (C and D) Quantification of dendritic spine density in juvenile (C) and adult mice (D). Juveniles: n_shControl_ = 38, n_shGluD1_ = 22, n_GluD1 OE_ = 26. Adults: n_shControl_ = 15, n_shGluD1_ = 13. (E) Segments of dendrites expressing shControl or shGluD1 along with PSD95.FingR-EGFP in juvenile mice. Dashed lines define the contours of tdTomato fluorescence. Scale bar: 2 μm. (F) Quantification of PSD-95 cluster density. n_shControl_ = 21, n_shGluD1_ = 24. (G) EGFP-gephyrin clusters in representative segments of dendrites expressing shControl, shGluD1, or shGluD1 together with shGluD1-resistant GluD1^∗^ in juvenile mice. Scale bar: 2 μm. (H and I) Quantifications of gephyrin cluster density in juvenile (H) and adult mice (I). Juveniles: n_shControl_ = 41, n_shGluD1_ = 30, n_shGluD1 + GluD1^∗^_ = 32. Adults: n_shControl_ = 11, n_shGluD1_ = 30. (J) Segments of dendrites illustrating the effects of Crispr-mediated *Grid1* knockout (KO) and GluD1 OE on gephyrin cluster density. Ctrl sgRNA, control sgRNA; KO sgRNA, *Grid1*-targeting sgRNAs. Scale bar: 2 μm. (K) Quantification of gephyrin cluster density in the conditions described above. n_Ctrl sgRNA_ = 22, n_KO sgRNA_ = 27, and n_GluD1 OE_ = 26. Statistics: bars indicate mean ± SEM, ns: p > 0.05, ^∗∗^p < 0.01, ^∗∗∗^p < 0.001. One-way ANOVA test followed by Tukey’s post test in (C) and (H); unpaired t test in (D), (F), and (K); and Mann-Whitney test for the comparison of GluD1 OE in K with corresponding control (shControl) in (H).

In order to assess the role of GluD1 in synaptic development, we used cortex-directed IUE at embryonic day (E)15.5. IUE at E15.5 allows the sparse and specific modification of layer 2/3 CPNs in their intact environment and the dissection of cell autonomous mechanisms operating at synapses *in vivo* (Figure 1A). We analyzed the consequences of GluD1 depletion or overexpression on excitatory and inhibitory synapses formed on oblique apical dendrites of layer 2/3 CPNs of the somato-sensory cortex using a morphometric approach (Figure 1A). We first used dendritic spines, the postsynaptic site of the majority of excitatory synaptic inputs in the brain (Bourne and Harris, 2008, Yuste, 2013), and clusters of PSD-95, a major scaffolding protein of excitatory synapses (Sheng and Hoogenraad, 2007), as a proxy for excitatory synapses (Figure 1B). We found that GluD1 depletion using short hairpin RNAs (shRNAs) (shGluD1; Figure S1A) did not affect the density of dendritric spines in juvenile (postnatal day [P]20–22) or adult (P > 69) mice (102% ± 3% and 105% ± 5% of control in juvenile and adult neurons respectively; Figures 1B–1D) or the density of endogenous PSD-95 clusters visualized using EGFP-tagged fibronectin intrabodies generated with mRNA display (FingR) (Gross et al., 2013) (94% ± 5% of control; Figures 1E and 1F). GluD1 overexpression, however, decreased spine density to 75% ± 4% of the control value (Figures 1B and 1C). These results suggest that GluD1 is not necessary for the formation or maintenance of excitatory synapses in layer 2/3 CPNs, though GluD1 may constrain their number if upregulated.

We then assessed the role of GluD1 at inhibitory synapses. To that aim, we expressed small amounts of fluorescent (EGFP-tagged) gephyrin (Figure 1A), the core component of inhibitory postsynaptic scaffolds (Krueger-Burg et al., 2017, Tyagarajan and Fritschy, 2014). This approach has been shown to reliably label inhibitory synaptic contacts without affecting synaptic development or inhibitory neurotransmission (Chen et al., 2012, Fossati et al., 2016, van Versendaal et al., 2012). In juvenile mice, GluD1 knockdown (KD) using shRNAs decreased the density of gephyrin clusters compared to control neurons (77% ± 4% of control; Figures 1G and 1H; Figure S2). Normal gephyrin cluster density was rescued by co-electroporating shGluD1 with a KD-resistant GluD1 construct (GluD1^∗^; Figures 1G and 1H; Figure S1A). Remarkably, the decrease in gephyrin cluster density induced by GluD1 KD was maintained in adults (79% ± 4% of control; Figure 1I), indicating that the loss of inhibitory synapses was not compensated over time. To further substantiate the role of GluD1 at inhibitory synapses, we knocked out *grid1* in single cells using the CRISPR-Cas9 system. We expressed the enhanced specificity espCas9(1.1) (Slaymaker et al., 2016) and a combination of two guide RNAs (gRNAs) using IUE. In *grid1* knockout (KO) neurons, the density of gephyrin clusters was decreased by 22% ± 5% compared to control neurons expressing espCas9(1.1) with mismatched gRNAs (Figures 1J and 1K), which is consistent with GluD1 KD experiments with shRNAs. In line with these results, GluD1 overexpression increased the density of gephyrin clusters along dendrites by 33% ± 4% (Figures 1J and 1K).

**Figure 2:**
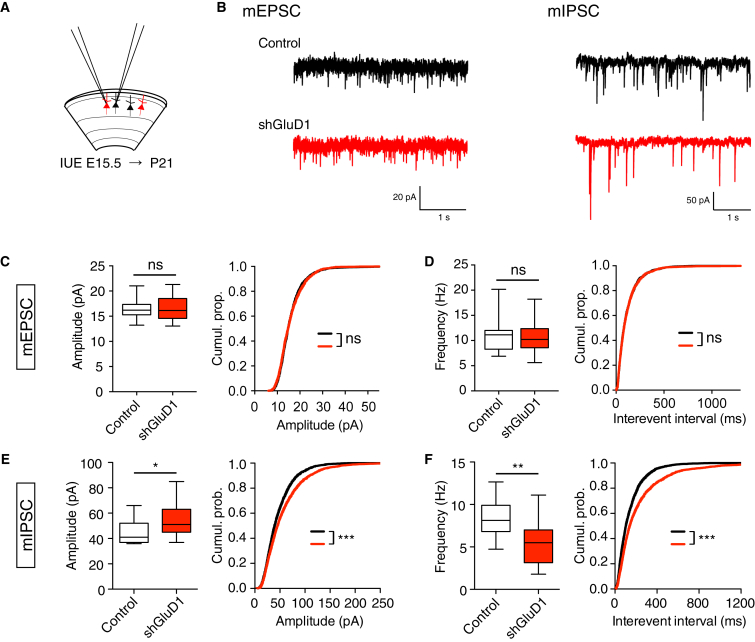
GluD1 Regulates Inhibitory Synaptic Transmission (A) Schematic: recording of electroporated layer 2/3 CPN expressing shGluD1 with tdTomato (red) and neighboring control neuron (black) from juvenile mouse brain slice. (B) Representative traces of mEPSCs and mIPSCs in control and shGluD1-electroporated neurons. (C and D) Quantification of mEPSC amplitude (C) and frequency (D). Boxplots (left) show the distribution of the mean value per cell. n = 14 in both conditions. Cumulative distributions (right) of the amplitudes and interevent intervals of the first 200 events of each cell. (E and F) Same as (C) and (D) for mIPSCs. n = 15 in both conditions. Statistics: ns p > 0.05, ^∗^p < 0.05, ^∗∗^p < 0.01, ^∗∗∗^p < 0.001, Mann-Whitney test.

To test the physiological consequences of GluD1 inactivation on synaptic transmission, we performed whole-cell patch-clamp recording in electroporated GluD1-depleted neurons and in neighboring non-electroporated control neurons (Figure 2A). We compared miniature excitatory and inhibitory postsynaptic currents (mEPSCs and mIPSCs, respectively) in brain slices from juvenile mice (Figure 2B). In line with the morphological data, GluD1 KD did not affect the amplitude or the frequency of mEPSCs (98% ± 8% and 100% ± 4% of control, respectively) (Figures 2B–2D). In contrast, GluD1 KD slightly increased the amplitude of mIPSCs and decreased their frequency by ≈35% (Figures 2B, 2E, and 2F), which is consistent with the reduced gephyrin cluster density observed in the oblique dendrites of GluD1 KD and KO neurons. We conclude that GluD1 in CPNs is selectively required for the formation of inhibitory synapses. It regulates both the assembly of the gephyrin-based postsynaptic scaffold and inhibitory synaptic transmission.

### GluD1 Localizes to Inhibitory Postsynaptic Sites

**Figure 3:**
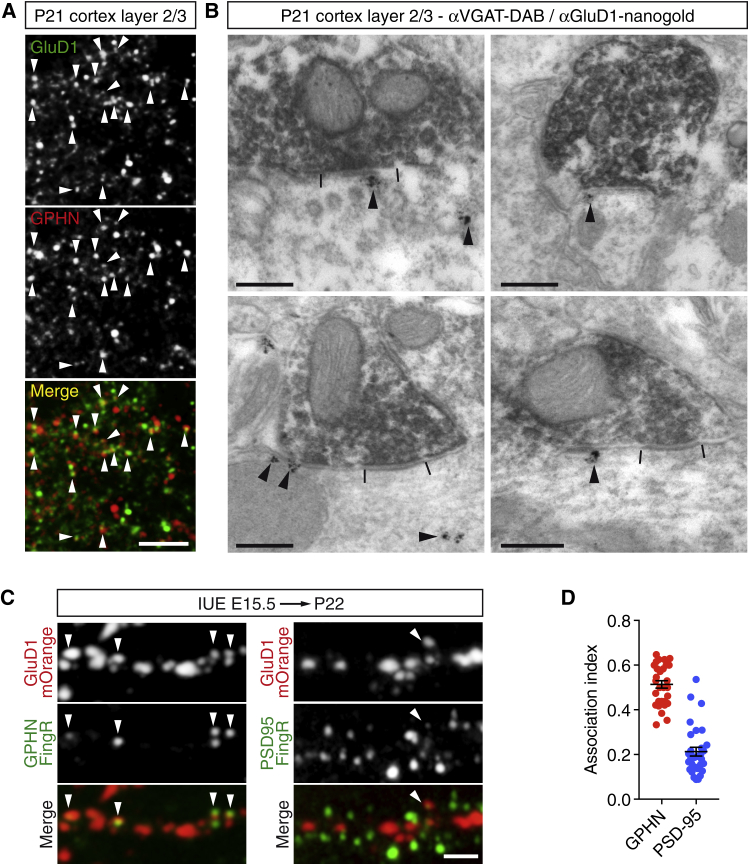
GluD1 Localizes to Inhibitory Synapses (A) Representative immunofluorescence image of P21 cortical slices stained with anti-GluD1 (green) and anti-gephyrin (red) antibodies. Arrowheads indicate the association between GluD1- and GPHN-positive clusters. Scale bar: 5 μm. (B) Electron micrographs showing GluD1 (stained using nanogold particles and silver enhancement) and VGAT (revealed with diaminobenzidine-positive) immunoreactivity in P21 layer 2/3 cortices. As indicated by the arrowheads, GluD1 was detected in front of inhibitory presynaptic terminals (top left image), or lateral to the active zone (delimited by two bars, other images) and in intracellular compartments (left images). Scale bars: 250 nm. (C) Segments of oblique apical dendrites from P22 neurons *in utero* electroporated with GluD1-mOrange and GPHN.FingR-EGFP or PSD95.FingR-EGFP. Arrowheads display the association between GluD1 and indicated synaptic markers. Scale bar: 2 μm. (D) Scatterplot showing the fraction of gephyrin and PSD-95 clusters associated with GluD1-mOrange puncta (association index). Gephyrin, n = 31; PSD-95, n = 30. Bars indicate mean ± SEM.

It is unexpected for a member of the iGluR family to control the formation of inhibitory synapses. Therefore, we asked whether GluD1 accumulates at inhibitory synapses. To answer this question, we performed immunohistochemistry in brain slices from juvenile mice. GluD1 fluorescent puncta were frequently associated with gephyrin clusters in the upper layers of the somato-sensory cortex (Figure 3A). To determine the precise subcellular localization of GluD1, we employed immuno-electron microscopy (EM). In cortical layer 2/3, inhibitory synapses represent only 10% of the total number of synapses, and they are “symmetrical” when observed in EM, meaning that they do not show the electron-dense post-synaptic differentiation facilitating the detection of excitatory synapses. To unambiguously identify inhibitory synapses, we performed double immunostaining of the vesicular GABA transporter (VGAT) and GluD1 (Figure 3B). We used diaminobenzidine (DAB) to reveal VGAT. DAB oxidation forms electron-dense precipitates that largely stained VGAT-positive inhibitory presynaptic terminals. To visualize and precisely localize GluD1 in cortical tissue, we used nanogold particles and silver enhancement. Nanogold particles corresponding to GluD1 were detected in front of VGAT-positive presynaptic terminals and in intracellular compartments in dendrites (Figure 3B). Within synapses, GluD1 was frequently observed in postsynaptic membrane domains located at the edge or in the periphery of the active zone, which is consistent with the distribution profile of other synaptic adhesion molecules (Triller and Choquet, 2003, Uchida et al., 1996). To quantify the proportion of synapses containing GluD1, we electroporated mOrange-tagged GluD1 together with EGFP-tagged GPHN or PSD95 FingRs and a soluble blue fluorescent protein to visualize neuronal morphology (Figure 3C, blue filler not shown). In oblique apical dendrites of layer 2/3 CPNs, ≈50% of inhibitory synapses contained GluD1 (Figure 3D). In contrast, GluD1 was rarely associated with excitatory synapses (21% ± 2%) (Figure 3D). Collectively, these results demonstrate the presence of GluD1 at inhibitory synapses and indicate that GluD1 directly operates at inhibitory synapses.

### Inhibitory Synapse Formation Requires GluD1 Binding to Cbln and Activation by Glycine or D-Serine

**Figure 4:**
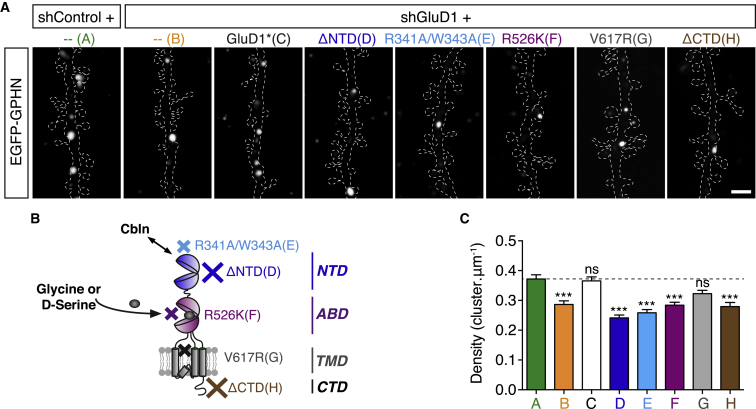
*In Vivo* Structure-Function Analysis of GluD1 Function at Inhibitory Synapses (A) EGFP-gephyrin clusters in representative segments of oblique dendrites in control condition (shControl) or after *in utero* replacement of endogenous GluD1 with indicated mutants in P20–22 mice. Dashed lines, dendritic contours based on tdTomato fluorescence. Scale bar: 2 μm. (B) Schematic of a GuD1 subunit and localization of the indicated mutations. GluD1 receptors interact with Cbln bound to presynaptic neurexin via their NTD and use glycine or D-serine as agonists. NTD, N-terminal domain; ABD, agonist-binding domain; TMD, transmembrane domain; CTD, C-terminal domain. (C) Quantification of GPHN cluster density in conditions represented in (A). Histogram represents means ± SEM. Data corresponding to shControl, shGluD1, and shGluD1 + GluD1^∗^ are the same as in Figure 1G. n_shControl_ = 41, n_shGluD1_ = 30, n_shGluD1 + GuD1^∗^_ = 32, n_shGluD1 + GluD1 ΔNTD_ = 30, n_shGluD1 + GluD1 R3341A/W343A_ = 32, n_shGluD1 + GluD1 R526K_ = 30, n_shGluD1 + GluD1 V617R_ = 29, n_shGluD1 + GluD1 ΔCTD_ = 28. ns p > 0.05, ^∗∗∗^p < 0.001, determined by one-way ANOVA test followed by Tukey’s post test.

To determine the molecular basis for GluD1-mediated regulation of inhibitory synapses, we took advantage of the recent crystallographic analysis of the interactions between GluD, Cbln, and neurexin and the abundant literature on the structure/function of GluD2 (Cheng et al., 2016, Elegheert et al., 2016, Kakegawa et al., 2007, Kakegawa et al., 2009, Kuroyanagi and Hirano, 2010, Yuzaki and Aricescu, 2017). GluDs, as all members of the iGluR family, are tetrameric receptors (Traynelis et al., 2010). Each subunit has a modular architecture. The extracellular region contains a distal N-terminal domain (NTD), followed by an agonist-binding domain (ABD). The NTD of the GluD receptor interacts with the extracellular scaffolding protein Cbln (Matsuda et al., 2010, Uemura et al., 2010), and their ABD binds to glycine and D-serine (but not glutamate) (Naur et al., 2007), as in some N-methyl-D-aspartate (NMDA) receptor subunits (Paoletti et al., 2013). The transmembrane domain (TMD) lines the pore of the ion channel. Finally, GluDs contain a C-terminal cytoplasmic domain (CTD) regulating their trafficking and intracellular interactions (Kakegawa et al., 2008, Kohda et al., 2013). To assay the functional importance of known domains or molecular interactions, we generated key mutant forms of GluD1 (Figure 4B). We then used an *in utero* gene replacement strategy to inactivate endogenous *grid1* with shRNAs and replace it with individual mutant forms *in vivo* and throughout development (Fossati et al., 2016). This strategy prevents the formation of heteromers between wild-type and mutant subunits of GluD1, which could mask or attenuate some phenotypes. Importantly, all mutants were properly trafficked to the cell surface (Figure S3A).

We first examined whether GluD1 function involves *trans*-synaptic interaction via Cbln. To that aim, we replaced endogenous GluD1 with a ΔNTD mutant lacking the whole NTD. In juvenile mice, gephyrin cluster density in neurons expressing this mutant was lower than in control (65% ± 4% of control), suggesting that the NTD is critical for GuD1 function (Figures 4A and 4C). We then specifically disrupted GluD1 interaction with Cbln by introducing two point mutations in the NTD (R341A/W343A, Figure S3B; residues corresponding to R345 and W347 in GluD2; Elegheert et al., 2016). Replacement of GluD1 with the R341A/W343A mutant also led to a lower density of gephyrin clusters (69% ± 4% of control; Figures 4A and 4C), indicating that GluD1 interaction with the extracellular scaffolding protein Cbln is required for inhibitory synapse formation. Next, we tested whether the regulation of inhibitory synapses requires GluD1 gating by glycine/D-serine, ion-flux through GluD1 channel, and signaling via the CTD of the receptor. Replacement of GluD1 with a mutant containing an arginine to lysine substitution at position 526, which abolishes the affinity for glycine or D-serine (R526K mutant corresponding to position 530 in GluD2) (Kakegawa et al., 2009, Kakegawa et al., 2011, Naur et al., 2007), decreased the density of gephyrin clusters (71% ± 4% of control; Figures 4A and 4C), as observed after GluD1 KD. A similar effect was found with a mutant lacking the intracellular CTD (75% ± 5% of control; Figures 4A and 4C). In contrast, preventing ion flux through the pore with a single point mutation (V617R) (Ady et al., 2014, Kakegawa et al., 2007, Robert et al., 2002) did not interfere with the formation of inhibitory synapses (Figures 4A and 4C). Collectively, these results demonstrate that the control of inhibitory synapse formation by GluD1 in CPNs requires *trans*-synaptic interactions via Cbln and glycine/D-serine-dependent non-ionotropic postsynaptic mechanisms involving intracellular interactions via the C-terminal tail of the receptor.

### GluD1 Specifies Synapses between SST^+^ INs and Layer 2/3 CPNs

**Figure 5:**
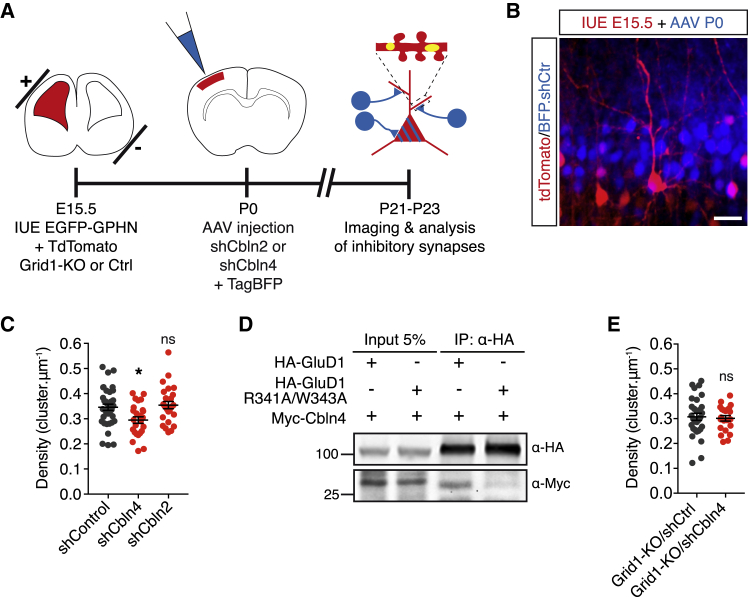
GluD1 Regulates Inhibitory Synaptogenesis via Cerebellin-4 (A) Schematic of the experimental workflow: neurons and inhibitory synapses were labeled using IUE at E15.5, which also allowed *grid1* knockout with Crispr. The lateral ventricle corresponding to the electroporated hemisphere was then injected with AAVs at P0 to knock down Cbln2 or Cbln4 with shRNAs. Inhibitory synapses were analyzed in juvenile mice. (B) Representative image of a cortical slice derived from a juvenile mouse. Electroporated layer 2/3 CPNs are labeled with tdTomato. Infected neurons are labeled with TagBFP. Scale bar: 20 μm. (C and E) Quantification of GPHN cluster density in neurons in the indicated conditions. n_shControl_ = 37, n_shCbln4_ = 25, n_shCbln2_ = 26, n_GluD1-KO/shCtr_ = 30, n_GluD1-KO/shCbln4_ = 22. Bars indicate mean ± SEM. ns p > 0.05, ^∗^p < 0.05, determined by one-way ANOVA test followed by Tukeys’s post test (C) or unpaired t test (E). (D) Coimmunoprecipitation (coIP) in HEK cells of Myc-Cbln4 with wild-type HA-GluD1 or HA-GluD1 containing R341A/W343A mutation in the Cbln binding site.

We wondered if GluD1 mediates the formation of inhibitory synapses between layer 2/3 CPNs and specific classes of INs. In the cortex, distinct subtypes of INs express distinct isoforms of Cblns, with SST^+^ INs in upper cortical layers expressing Cbln4 and vasoactive intestinal peptide-positive (VIP^+^) INs expressing Cbln2 (Paul et al., 2017, Tasic et al., 2016). Therefore, we tested if Cbln2 and Cbln4 regulate the density of inhibitory synapses. To that end, adeno-associated viral vectors (AAVs) carrying an shRNA directed against Cbln4, Cbln2, or a control shRNA (Figure S1D) were injected *in vivo* in the lateral ventricles of newborn pups previously electroporated *in utero* with EGFP-GPHN and tdTomato (Figures 5A and 5B). We then quantified the density of gephyrin clusters in sparse electroporated neurons surrounded by numerous infected cells (Figure 5B). In juvenile mice, Cbln4, but not Cbln2, inactivation significantly decreased the density of inhibitory synapses (85% ± 4% of control for shCbln4 and 102% ± 4% for shCbln2; Figure 5C). GluD1 interacted with Cbln4 (Figure 5D), and Cbln4 KD did not further decrease the density of gephyrin clusters in *grid1* KO neurons (98% ± 5% of Grid1-KO/shCtrl neurons; Figure 5E), indicating that Cbln4 operates via GluD1. The role of Cbln4 at inhibitory synapses between SST^+^ INs and CPNs has recently been characterized in more detail by Favuzzi et al. (2019). Taken together, these results indicate that GluD1 specifies inhibitory cortical connectivity by mediating synaptogenesis between Cbln4-expressing SST^+^ INs and CPNs. This is compatible with the partial colocalization of GluD1 and gephyrin (Figures 3C and 3D) in oblique apical dendrites, which are also contacted by other classes of interneurons (Fishell and Kepecs, 2019).

### Postsynaptic Signaling Controlling Inhibitory Synapse Formation

**Figure 6:**
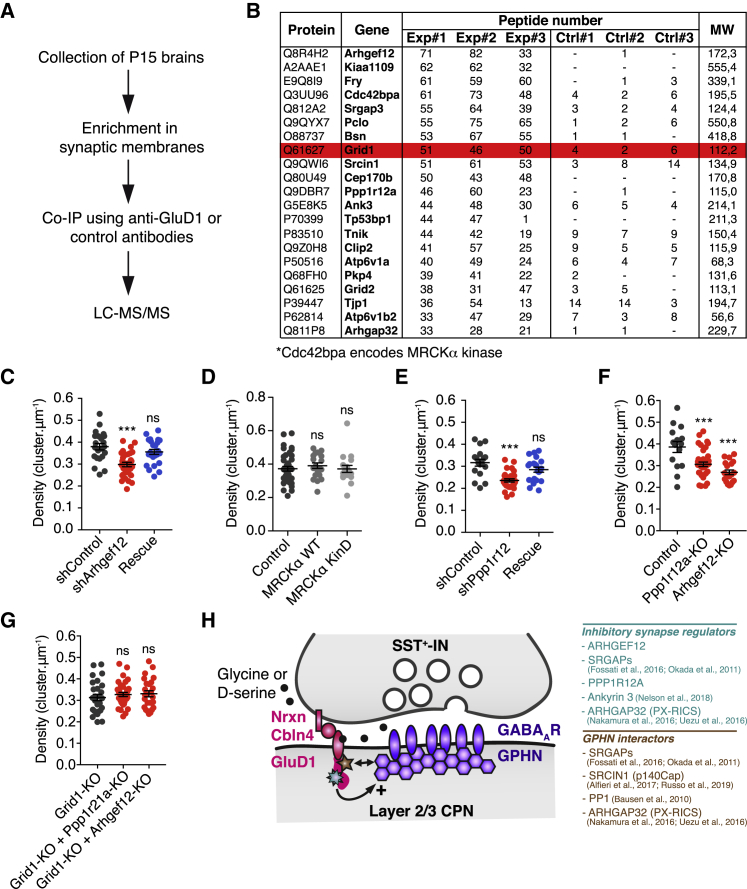
Signaling Pathways Regulated by GluD1 at Inhibitory Synapses (A) Schematic of the experimental workflow used for mass spectrometry analysis. CoIP, co-immunoprecipitation; LC-MS/MS, liquid chromatography followed by tandem mass spectrometry. (B) Table displaying the 20 most abundant GluD1-interacting proteins. Exp#1–#3 represent independent coIP replicates with anti-GluD1 antibody; Ctrl#1–#3 corresponds to the control coIPs with total rabbit IgG. Only proteins enriched at least three times in GluD1 coIPs are indicated. GluD1 (encoded by the gene *grid1*) is highlighted in red. (C–G) Quantification of GPHN cluster density in oblique dendrites of layer 2/3 CPNs *in utero* electroporated in the indicated conditions (juvenile stage). MRCKα KinD, MRCKα kinase-dead mutant; MRCKα WT, wild-type MRCKα. n_shControl (C)_ = 22, n_shArhgef12_ = 32, n_Rescue (C)_ = 24, n_Control (D)_ = 41, n _MRCKα WT_ = 20, n _MRCKα KinD_ = 18, n_shControl (E)_ = 17, n_shPpp1r12a_ = 30, n_Rescue (E)_ = 21, n_Control (F)_ = 15, n_Ppp1r12a-KO_ = 38, n_Arhgef12-KO_ = 19, n_Grid1-KO (G)_ = 27, n_Grid1-KO + Ppp1r12a-KO_ = 34, n_Grid1-KO + Arhgef12-KO_ = 25. Statistics: bars indicate mean ± SEM, ns p > 0.05, ^∗∗∗^p < 0.001 determined by one-way ANOVA test followed by Tukey’s post test in (D-G) and Kruskal-Wallis test followed by Dunn’s post test in (C). (H) Schematic illustrating GluD1 *trans*-synaptic signaling regulating inhibitory synapse development. See text for details.

To determine the postsynaptic mechanisms through which GluD1 regulates the formation of inhibitory synaptic machineries, we performed an unbiased proteomic screen aimed at identifying GluD1 interacting partners at synapses. We employed subcellular fractionations from P15 mouse brains to enrich our samples in proteins associated with synaptic membranes and efficiently immunoprecipitate GluD1 (Figure 6A). The proteins co-immunoprecipitated with GluD1 (gene name *Grid1*) were separated by liquid chromatography and identified using tandem mass spectrometry (LC-MS/MS) (Figures 6A and 6B; Table S1). We focused on the proteins that were the most represented in terms of the number of detections in LC-MS/MS biological triplicates relative to their molecular weight. GluD1 was strongly associated with regulators of GTPases (e.g., ARHGEF12 and SRGAP3) and regulators of protein phosphorylation (e.g., the serine/threonine phosphatase 1 regulatory subunit PPP1R12A and the serine/threonine protein kinase MRCKα, encoded *cdc42bpa*), pointing out the involvement of signaling pathways.

To determine the contribution of these proteins to the development of inhibitory synapses, we manipulated their expression *in vivo* using IUE. We first investigated the role of ARHGEF12 (also referred to as LARG), a guanine nucleotide exchange factor for RhoA (Chen et al., 1999). We generated an shRNA against *arhgef12* (shArhgef12) and an shRNA-resistant construct (ARHGEF12^∗^; Figure S1B). In juvenile mice, sparse *arhgef12* KD decreased the density of gephyrin clusters to 79% ± 3% of the control value (Figure 6C). Normal gephyrin cluster density was rescued by ARHGEF12^∗^ (94% ± 3%). These data identify ARHGEF12 as a new determinant of inhibitory synapse formation in the dendrites of CPNs. We next considered the role of Slit-Robo Rho GTPAse-activating proteins (SRGAPs). In LC-MS/MS, GluD1 was associated not only with SRGAP3, but also with SRGAP1 and SRGAP2 (Table S1). SRGAP3 and SRGAP2 have previously been shown to interact with gephyrin and regulate the development of inhibitory synapses in the hippocampus and the cortex, respectively (Fossati et al., 2016, Okada et al., 2011). We found that SRGAP2 inactivation decreases the cell surface expression of GluD1 in young (15 days *in vitro*), but not older (22–23 days *in vitro*), neurons (Figure S4), suggesting a developmental regulation of GluD1 trafficking.

We then assayed the role of MRCKα and PPP1R12A, two proteins likely to modulate the phosphorylation state of proteins implicated in building up inhibitory synapses. Neither the overexpression of a kinase-dead dominant-negative mutant of MRCKα (MRCKα KinD, with K106A substitution) (Leung et al., 1998) nor that of wild-type MRCKα (MRCKα WT) affected the density of gephyrin clusters (Figure 6D) in juvenile mice, indicating that MRCKα is not critical for the formation of inhibitory synapses. In contrast, depletion of PPP1R12A (also referred to as MYPT1), a targeting subunit of PP1, with shRNAs decreased the density of gephyrin clusters (74% ± 3% of the control), and normal gephyrin cluster density was rescued with the shRNA-resistant mutant PPP1R12A^∗^ (Figure 6E; Figure S1C), demonstrating that PPP1R12A is required for the formation of inhibitory synapses in dendrites. These data are in line with a previous study indicating that PP1 physically interacts with gephyrin and regulates the density of gephyrin clusters (Bausen et al., 2010).

Since both ARHGEF12 and PPP1R12A inactivation mimic GluD1 loss of function, our data suggest that GluD1 might signal through these two proteins to mediate the formation of inhibitory synapses. Using the CRISPR-Cas9 system combined with IUE to inactivate *grid1* (Figures 1J and 1K), *ppp1r12a*, and *arhgef12* (Figure 6F), we found that neither *grid1/arhgef12* double KO nor *grid1/ppp1r12a* double KO further reduced the density of inhibitory synapses compared to single *grid1* KO (104% ± 3% of Grid1-KO for Grid1-KO + Ppp1r12a-KO and 105% ± 4% of Grid1-KO for Grid1-KO + Arhgef12-KO; Figure 6G), indicating that GluD1 requires ARHGEF12 and PPP1R12A to operate at inhibitory synapses. Interestingly, other major partners of GluD1 (Figure 6B), such as the Rho GTPase-activating protein 32 (ARHGAP32/PX-RICS) (Nakamura et al., 2016, Uezu et al., 2016), SRCIN1 (p140cap) (Alfieri et al., 2017, Russo et al., 2019), and Ankyrin 3 (Ankyrin G) (Nelson et al., 2018), have been shown to interact with gephyrin and/or regulate inhibitory synaptogenesis. This supports the notion that GluD1 serves as a signaling hub for the formation and specification of inhibitory synapses (Figure 6H), and that the regulation of inhibitory synaptogenesis is a major function of GluD1 in the neocortex.

## Discussion

In the present study, we employed sparse *in vivo* molecular manipulations and proteomic approaches to characterize the role of GluD1 in synaptic development in layer 2/3 pyramidal neurons of the somato-sensory cortex. We demonstrate that GluD1 is a maverick among iGluRs, operating at inhibitory synapses rather than excitatory synapses. GluD1 is necessary for the formation of specific inhibitory synapses along dendrites and regulates GABAergic synaptic transmission accordingly. In the upper layers of the cortex, GluD1 is enriched in the postsynaptic membrane of inhibitory synapses, lateral to the active zone, where it establishes *trans*-synaptic interactions via Cbln4, an extracellular scaffolding protein secreted by SST^+^ INs (Favuzzi et al., 2019), which, in turn, binds to presynaptic neurexins (Yuzaki, 2017, Zhong et al., 2017). When interacting with Cbln4 and binding to glycine or D-serine, GluD1 activates postsynaptic signaling pathways that do not depend on ion flux through its channel, but it involves intracellular interactions via its C-terminal tail, organizing the assembly of inhibitory postsynaptic machineries at contact sites with SST^+^ INs. These interactions involve ARHGEF12 and PPP1R12A, two molecules required for GluD1 function at inhibitory synapses and probably other molecules implicated at GABAergic synapses.

### Region-Specific Function of GluD1 at Inhibitory Synapses

Although the repertoire of inhibitory synaptic proteins has recently expanded (Krueger-Burg et al., 2017), the molecular diversity of inhibitory synapses and the difficulty of investigating their biochemistry and their cell biology *in vivo* has obscured the mechanistic understanding of inhibitory synaptogenesis. Hence, few CAMs and signaling molecules have been shown to selectively control inhibitory synapse assembly (Krueger-Burg et al., 2017, Tyagarajan and Fritschy, 2014). These molecules show domain-specific functions at perisomatic (Früh et al., 2016, Poulopoulos et al., 2009), dendritic (Li et al., 2017), or axo-axonic synapses (Panzanelli et al., 2011). Their function also varies depending on brain areas. In particular, extensive studies of neuroligin 2 and collybistin (ARHGEF9), two proteins present in virtually all inhibitory synapses in the CNS, have highlighted fundamental differences between the cell biology of hippocampal and cortical inhibitory synapses (Gibson et al., 2009, Poulopoulos et al., 2009, Papadopoulos et al., 2007). Since the mechanisms of synaptogenesis in the forebrain are predominantly studied in hippocampal neurons, the molecular underpinning of inhibitory synapse formation in the cortex has remained enigmatic.

Previous *in vitro* hemi-synapse formation assays suggested a synaptogenic activity of GluD1 in cortical neurons (Ryu et al., 2012, Yasumura et al., 2012). However, it was unclear whether the synaptogenic activity was selective for inhibitory synapses (Yasumura et al., 2012) or common to excitatory and inhibitory synapses (Ryu et al., 2012), and the role of endogenous GluD1 in cortical neurons *in vivo* remained unclear (Gupta et al., 2015). Here, we used *in vivo* single cell approaches based on sparse IUE to manipulate GluD1 expression and function in isolated layer 2/3 CPNs in the intact brain. Targeting a specific cell type allowed us to investigate a relatively homogeneous population of neurons and dissect cell-autonomous mechanisms with a subcellular resolution in spatially identified synapses along the dendritic tree. Moreover, sparse *in vivo* manipulations help avoid compensatory and adaptive changes at the network level, which might occur in KO mouse models. Our results provide direct evidence that GluD1 is necessary for the formation of specific cortical inhibitory synapses *in vivo*. While we do not exclude that GluD1 could regulate some properties of excitatory synapses in the cortex, we clearly show that GluD1 is not required for their formation, which is consistent with a previous study (Gupta et al., 2015). Therefore, GluD1 function in CPNs starkly contrasts with GluD1 function in cerebellar INs (Konno et al., 2014) and hippocampal pyramidal neurons (Tao et al., 2018), where GluD1 is required for the formation of excitatory synapses. This raises fundamental questions on the molecular basis underlying the region-specific function of GluD1 at excitatory or inhibitory synapses and the synaptic dysfunction associated with GluD1 mutations in brain disorders (Cooper et al., 2011, Fallin et al., 2005, Glessner et al., 2009, Guo et al., 2007, Treutlein et al., 2009).

### An iGluR-Dependent Signaling Pathway at Inhibitory Synapses

It is unconventional for a member of the iGluR family to locate and operate at inhibitory synapses. We show that *trans*-synaptically engaged GluD1 binding to glycine or D-serine initiates postsynaptic signaling via non-ionotropic mechanisms, probably through conformational changes that are transmitted through the TMD, and controls intracellular interactions (Elegheert et al., 2016). Whether GluD1 binds to glycine or D-serine in CPNs is unknown, but both glycine and D-serine may contribute to the regulation of inhibitory synapse formation. Glycine is present in the extracellular space, where it activates extrasynaptic NMDA receptors (Papouin et al., 2012) and mediates tonic inhibition in layer 2/3 CPNs (McCracken et al., 2017, Salling and Harrison, 2014). In the brainstem and spinal cord, where glycine is a major inhibitory neurotransmitter and GluD1 is highly expressed (https://www.gtexportal.org/home/gene/GRID1), presynaptic release of glycine may directly regulate the formation and maintenance of inhibitory synapses. D-serine is also present in the extracellular environment. It is synthesized through conversion of L-serine by serine racemase and released at least by the neuronal alanine-serine-cysteine transporter 1 (Asc-1) (Rosenberg et al., 2013). Ambient D-serine level is regulated by excitatory glutamatergic activity (Van Horn et al., 2017, Ma et al., 2014), and low D-serine levels are associated with epilepsy (Klatte et al., 2013) and schizophrenia-like behaviors (Ma et al., 2013), consistent with defects in synaptic inhibition and GluD1 function. So far, in the forebrain, the role of D-serine and, to some extent, glycine has been envisioned through the activation of NMDA receptors (Oliet and Mothet, 2009). The role of GluD1 in establishing the equilibrium between excitation and inhibition and the requirement of GluD1 activation by glycine/D-serine suggests that some functions initially attributed to NMDA receptors might instead depend on GluD1 signaling.

Proteomic and functional analyses of GluD1 interactome allowed us to identify signaling pathways controlling the postsynaptic organization of inhibitory machineries. We found that two major partners of GluD1, ARHGEF12 and PPP1R12A, are necessary for inhibitory synapse formation in layer 2/3 CPNs. Double inactivation experiments showed that ARHGEF12 and PPP1R12A operate in the same pathway as GluD1 and therefore also contribute to the specification of inhibitory connectivity between SST^+^ INs and layer 2/3 CPNs. ARHGEF12 contains a Dbl-homology (DH) domain mediating guanosine diphosphate (GDP)/guanosine triphosphate (GTP) exchange activity and a pleckstrin-homology (PH) domain, which binds phosphoinositides and regulates its membrane targeting (Hyvönen et al., 1995). ARHGEF12 also contains an N-terminal PDZ domain, which may interact with the C-terminal PDZ-binding motif of GluD1, and a regulator of G-protein signaling-like (RGSL) domain. Although further experiments are needed to determine how ARHGEF12 contributes to inhibitory synaptic development, one possibility is that ARHGEF12 links *trans*-synaptic interaction with phosphoinositide and G-protein signaling to mediate inhibitory synaptogenesis. Furthermore, our data on PPP1R12A suggest that targeting PP1 to GluD1-mediated contact sites between INs and pyramidal neurons and locally regulating the post-translational state of inhibitory synaptic components is critical to initiate or promote postsynaptic assembly. This is congruent with previous studies showing that PP1 associates with gephyrin and the beta-3 subunit of GABA_A_ receptors (GABA_A_Rs) (Bausen et al., 2010, Pribiag and Stellwagen, 2013) and that pharmacological inhibition of PP1 induces a loss of gephyrin clusters (Bausen et al., 2010). Among the most represented interactors of GluD1 we identify here, Ankyrin 3, ARHGAP32/PX-RICS, SRCIN1/p140Cap, and SRGAPs were previously implicated at inhibitory synapses. Ankyrin 3 interacts with GABA_A_R-associated protein (GABARAP) and contributes to stabilization of GABA_A_Rs in the postsynaptic membrane (Nelson et al., 2018). ARHGAP32 interacts with gephyrin, and its inactivation impairs GABA_A_R trafficking at synapses (Nakamura et al., 2016, Uezu et al., 2016). SRCIN1, SRGAP3, and SRGAP2 also associate with gephyrin and regulate GABAergic synaptogenesis (Alfieri et al., 2017, Fossati et al., 2016, Okada et al., 2011, Russo et al., 2019). Therefore, GluD1 trans-synaptic signaling provides local regulation of protein phosphorylation and GTPase activity and allows the recruitment of synaptic molecules for the assembly of inhibitory postsynaptic machineries in an input-specific and agonist-dependent manner. Interestingly, in young neurons, GluD1 expression at the cell surface was decreased by SRGAP2 inactivation. SRGAP2, and by homology SRGAP3, is inhibited by the human-specific protein SRGAP2C (Charrier et al., 2012, Coutinho-Budd et al., 2012). This regulation could contribute to the delay of the development of inhibitory synapses in human neurons (Fossati et al., 2016) and modify inhibitory circuitry to accommodate a greater diversity of IN subtypes (Defelipe, 2011). Understanding the diversity of *trans*-synaptic interactions and signaling pathways coordinating the establishment of neocortical inhibitory circuitry, their interplay, their evolution in human, and their dysregulations in brain disorders will be a fertile area for future research.

## STAR★Methods

### Key Resources Table

**Table:** 

REAGENT or RESOURCE	SOURCE	IDENTIFIER
**Antibodies**		

Mouse anti-HA	Covance	Cat# MMS-101P; RRID:AB_2314672
Rabbit anti-GFP	Thermo Fisher Scientific	Cat# A-6455; RRID:AB_221570
Rabbit anti-Myc	Cell Signaling Technology	Cat# 2272; RRID:AB_10692100
Mouse anti-Myc	Cell Signaling Technology	Cat# 2276; RRID:AB_331783
Rabbit anti-GluD1	Hepp et al., 2015	N/A
Mouse anti-gephyrin	Synaptic Systems	Cat# 147 011; RRID:AB_887717
Rabbit anti-Arhgef12	Thermo Fisher Scientific	Cat# PA5-39418; RRID:AB_2556008
Rabbit anti-Ppp1r12a	Thermo Fisher Scientific	Cat# PA5-17164; RRID:AB_10978517
Rabbit anti-RFP	Rockland	Cat# 600-401-379; RRID:AB_2209751
Rabbit anti-Srgap2	Charrier et al., 2012	N/A
Rabbit anti-GAPDH	Synaptic Systems	Cat# 247 002; RRID:AB_10804053
Mouse anti-VGAT	Synaptic Systems	Cat# 131 011; RRID:AB_887872
Goat anti-mouse HRP	Jackson ImmunoResearch Labs	Cat# 115-035-003; RRID:AB_10015289
Goat anti-rabbit HRP	Jackson ImmunoResearch Labs	Cat# 111-035-144; RRID:AB_2307391
Goat anti-rabbit Alexa 488	Thermo Fisher Scientific	Cat# A27034; RRID:AB_2536097
Goat anti-mouse Cy3	Jackson ImmunoResearch Labs	Cat# 115-165-003; RRID:AB_2338680
Goat biotinylated anti-mouse	Vector Laboratories	Cat# BA-9200; RRID:AB_2336171
Nanogold goat anti-rabbit	Nanoprobes	Cat# 2003; RRID:AB_2687591

**Bacterial and Virus Strains**		

AAV2/1 H1-shControl.Syn-TagBFP	This paper	N/A
AAV2/1 H1-shCbln2.Syn-TagBFP	This paper	N/A
AAV2/1 H1-shCbln4.Syn-TagBFP	This paper	N/A
Lentivirus H1-shControl.Syn-EGFP	This paper	N/A
Lentivirus H1-shGluD1.Syn-EGFP	This paper	N/A
Lentivirus H1-shPpp1r12a.Syn-EGFP	This paper	N/A
Lentivirus H1-shPpp1r12a.Syn-EGFP	This paper	N/A
Lentivirus H1-shSrgap2.Syn-EGFP	This paper	N/A

**Chemicals, Peptides, and Recombinant Proteins**		

Tetrodotoxin	Abcam	Cat# ab120055
NBQX	Abcam	Cat# ab120046
D-AP5	Abcam	Cat# ab120003
Gabazine	Abcam	Cat# ab120042

**Experimental Models: Cell Lines**		

HEK293T	ATCC	Cat# CRL-1573; RRID:CVCL_0045

**Experimental Models: Organisms/Strains**		

Mouse: Swiss/CD-1	Janvier Labs	N/A
Mouse: C57BL/6J	Janvier Labs	N/A

**Oligonucleotides**		

shRNA oligos, see methods detail	This paper	N/A
gRNA oligos, see methods detail	This paper	N/A

**Recombinant DNA**		

pCAG HA-GluD1	This paper	N/A
pCAG HA-GluD1-mOrange	This paper	N/A
pCAG_PSD95.FingR-EGFP-CCR5TC	Gross et al., 2013	RRID:Addgene_46295
pCAG_GPHN.FingR-EGFPCCR5TC	Gross et al., 2013	RRID:Addgene_46296
Mouse Ppp1r12a cDNA	Dharmacon	Cat# MMM1013-211691718
Human MRCKα cDNA	Dharmacon	Cat# MHS6278-213663984
pCDNA3 mRFP-ARHGEF12	Bodmann et al., 2017	N/A
Mouse Cbln2 cDNA	Dharmacon	Cat# MMM1013-202798518
Mouse Cbln4 cDNA	Dharmacon	Cat# MMM1013-202798044
pCAG 3xMyc-MRCKα	This paper	N/A
pCAG 3xMyc-Ppp1r12a	This paper	N/A
pCAG ARHGEF12	This paper	N/A
pCAG Myc-Cbln2	This paper	N/A
pCAG Myc-Cbln4	This paper	N/A
pH1SCV2 vectors	Charrier et al., 2012	N/A
pH1SCTdT2 vectors	Fossati et al., 2016	N/A
Lenti H1-shRNA.Syn-EGFP vectors	This paper	N/A
pAAV H1-shRNA.Syn-TagBFP vectors	This paper	N/A
eSpCas9(1.1) vectors	Slaymaker et al., 2016	RRID:Addgene_71814

**Software and Algorithms**		

GPP sgRNA Designer	Broad Institute	https://portals.broadinstitute.org/gpp/public/analysis-tools/sgrna-design
myProMS v3.6	Poullet et al., 2007	http://bioinfo-out.curie.fr/myproms/proms.html
Fiji	Schindelin et al., 2012	https://fiji.sc/; RRID:SCR_002285
pCLAMP10	Molecular Devices	RRID:SCR_011323
Prism 7	GraphPad	RRID:SCR_002798

### Lead Contact and Materials Availability

Further information and requests for resources and reagents should be directed to and will be fulfilled by the Lead Contact, Cécile Charrier (cecile.charrier@ens.fr).

### Experimental Model and Subject Details

#### Animals

All animals were handled according to French and EU regulations (APAFIS#1530-2015082611508691v3). In utero electroporations were performed on pregnant Swiss females at E14.5-15.5 (Janvier labs). For viral injections in the lateral ventricles, newborn pups (P0) of undetermined sex were used. Primary cultures were prepared from timed pregnant C57BL/6J mice at E18.5 (Janvier labs). Juveniles correspond to mice between P20 and P22. Adults correspond to mice between P69 and P75. Mice were maintained in a 12 hr light/dark cycle with unlimited access to food and water.

#### Primary cultures of mouse cortical neurons

Primary cultures were performed as described previously (Charrier et al., 2012) with few modifications. After dissection and dissociation of mouse cortices from E18.5 embryos, neurons were plated on glass coverslips coated with poly-D-ornytine (80 μg/ml, Sigma) in MEM supplemented with sodium pyruvate, L-glutamine and 10% horse serum. Medium was changed 2-3 h after plating with Neurobasal supplemented with L-glutamine (2 mM), B27 (1X) and penicillin (2.5 units/ml) - streptomycin (2.5 μg/ml). Then, one third of the medium was changed every 5 days. Unless otherwise indicated, all products were from Life Technologies. Cells were maintained at 37°C in 5% CO2 until use.

#### HEK293T cells

HEK293T (CRL-1573 from ATCC) cells were cultured according to suggested protocols. Briefly, cells were maintained in DMEM (GIBCO) supplemented with 10% fetal bovine serum (GIBCO) and 1% Penicillin- Streptomycin (GIBCO) at 37°C, 5% CO2, and passaged by trypsin/EDTA digestion (GIBCO) upon reaching confluency.

### Method Details

#### Plasmids for protein expression

EGFP-GPHN was previously described (Fossati et al., 2016) and it was used to visualize inhibitory synapses. HA-tagged mouse *grid1* (gift from Ludovic Tricoire, IBPS, Paris, France) was inserted into pCAG vector by PCR between XhoI and BsrGI to obtain pCAHA GluD1 or between XhoI and KpnI to obtain pCAHA GluD1-EGFP. EGFP was then replaced by mOrange between KpnI and NotI to generate pCAHA GluD1-mOrange. pCAG_PSD95.FingR-EGFP-CCR5TC and pCAG_GPHN.FingR-EGFP-CCR5TC were purchased from Addgene (plasmids #46295 and #46296, respectively). Mouse *ppp1r12a* (GenBank: BC125381, cDNA clone MGC:159084 IMAGE:40129896) and human *CDC42BPA* (encoding MRCKα, GenBank: BC136333, cDNA clone MGC:167943 IMAGE:9020320) cDNAs were obtained from Dharmacon and subcloned by PCR into pCAG vector between AgeI and BsrGI or AgeI and NotI, respectively. Both constructs were Myc-tagged by inserting a DNA cassette containing a start codon and 3x-Myc between AgeI and KpnI. Human *ARHGEF12* (gift from Moritz Bünemann, Marburg University, Germany) was inserted into pCAG vector by PCR between AgeI and BsrGI. Indicated mutations were introduced in *grid1*, *CDC42BPA*, *ppp1r12a*, and *ARHGEF12* using the QuickChange mutagenesis kit (Agilent). Mouse *cbln2* (GenBank: BC055682, cDNA clone MGC:66500 IMAGE:6412317) and *cbln4* (GenBank: BC094540, cDNA clone MGC:106619 IMAGE:5708067) cDNAs were obtained from Dharmacon and subcloned by PCR into pCAG vector between AgeI and NotI. The three constructs were Myc-tagged at the N terminus by inserting a DNA cassette containing a start codon, the signal sequence of Cbln1 (for Cbln2) or of Cbln4 (for Cbln4) and one Myc tag between EcoRI and AgeI.

#### shRNA and Crispr constructs and shRNA validation

For in utero knockdown experiments with shRNAs, we used the previously described pH1SCV2 and pH1SCTdT2 vectors (Charrier et al., 2012, Fossati et al., 2016). An H1 promoter drives the expression of the shRNA and a CAG promoter that of myristoylated Venus (mVenus) or TdTomato, respectively. The vector pH1SCV2 was used for dendritic spine analysis, pH1SCTdT2 was co-expressed with EGFP-GPHN to analyze inhibitory synapses. For shRNA validation on endogenous mouse *grid1*, *arhgef12* and *ppp1r12a,* we used a lentiviral vector carrying the H1 promoter to drive shRNA expression and the synapsin promoter to drive EGFP expression (Fossati et al., 2016). For AAV-mediated *in vivo* knockdown of *cbln2* and *cbln4* the plasmid pAAV-eIF1α-tdTomato-WPRE-pGHpA (Addgene, plasmid #67527; Wertz et al., 2015) was modified as follows: a DNA cassette containing the shRNA with the H1 promoter and EGFP with the synapsin promoter was amplified by PCR from the lentiviral vector described above and inserted into pAAV-eIF1α-tdTomato-WPRE-pGHpA between MluI and EcoRI. EGFP was then replaced by TagBFP between EcoRI and NheI. AAVs (serotype 2/1) were produced by the Centre of vector production of INSERM (CPV, UMR1089, Nantes, France). Control shRNA (shControl) was described previously (Charrier et al., 2012). The following shRNAs targeted the corresponding seed sequences: shGluD1: 5′-GAAGATAGCTCAAATCCTTAT-3′; shARHGEF12: 5′-GCAGCTGTTTCCAGAGCATTG-3′; shPpp1r12a: 5′-GCTGAAATCAGTGCGTCTAAA-3′; shCbln2: 5′- GCTTAATGCAGAATGGCTACC-3′; shCbln4: 5′-GCCGTTCTGCTGATTCTAGTG-3′. ShRNAs were validated as previously described (Charrier et al., 2012). Briefly, HEK293T cells were co-transfected with mouse HA-GluD1, Myc-Ppp1r12a, Myc-Cbln2, Myc-Cbln4 or human RFP-ARHGEF12 together with the corresponding shRNA at 1:2 ratio. Two days after transfection, cells were collected and lysed in RIPA buffer (150 mM NaCl, 1.0% NP-40, 0.5% sodium deoxycholate, 0.1% SDS, 50 mM Tris, pH 8.0, Sigma-Aldrich) and further processed for western blot analysis of the relative protein expression levels. The knockdown of endogenous *grid1*, *arhgef12* and *ppp1r12a* was further validated in primary cultures of cortical neurons infected with lentiviral vectors. Neurons infected at DIV (days *in vitro*) 4 were harvested at DIV21 and lysed in RIPA buffer under agitation for 1 h at 4°C. 20 μg of total proteins for neurons infected with shGluD1 and shArhgef12 or 40 μg of total proteins for neurons infected with shPpp1r12a were separated by SDS-PAGE and further processed for western blot analysis. For rescue experiments, four point silent mutations were introduced in *grid1* (c1193t_t1194a_c1195 g_a1196c), *ppp1r12a* (c1355t_a1356t_g1357c_t1358a) and *ARHGEF12* (g1119a_a1120t_g1121c_c1122a) to resist to shRNA-mediated knockdown (mutants named GluD1^∗^, Ppp1r12a^∗^ and ARHGEF12^∗^, respectively). To knock out *grid1*, *arhgef12* and *ppp1r12a* with Crispr, we used an engineered spCas9 with enhanced specificity (espCas9(1.1), Addgene plasmid #71814) (Slaymaker et al., 2016). gRNAs were designed using the prediction software: https://portals.broadinstitute.org/gpp/public/analysis-tools/sgrna-design. Each gene was knocked-out using two gRNAs that were encoded by the same plasmid together with the espCas(1.1). A DNA cassette containing the U6 promoter and one gRNA was inserted between XbaI and and KpnI into espCas9(1.1) plasmid carrying a second gRNA. *grid1*: 5′-GGCCAATAATCCGTTCCAGG-3′ (targets exon 2) and 5′-GAAACTCCATAACCCCTGTG-3′ (targets exon 8); *arhgef12*: 5′-GTCTACTATCACGGACAGGT-3′ (targets exon 1) and 5′-GGCATCACCTAATGGCCTGG-3′ (targets exon 11); *ppp1r12a*: 5-‘GGTGAAGCGCCAGAAGACCA-3′ (targets exon 1) and 5′-GTGTTGATATAGAAGCGGCT-3′ (targets exon 4).

#### Lentivirus production and infection

48 h after transfection of HEK293T cells, the viral supernatant was collected, centrifuged at 3,000 g for 5 min at 4°C to remove cell debris, and ultracentrifuged at 25,000 g for 2 h on a 20% sucrose cushion. Viral pellets were resuspended in sterile PBS, aliquoted and stored at −80°C. When indicated, cortical neurons were infected 4 days after plating with concentrated lentiviruses driving the expression of shRNA and EGFP.

#### In utero electroporation, AAV injection and slice preparation

In utero electroporation was performed as previously described (Fossati et al., 2016). Pregnant Swiss females at E14.5-15.5 (Janvier labs) were anesthetized with isoflurane (3.5% for induction and 2% during the surgery) and subcutaneously injected with 0.1 mg/kg of buprenorphine for analgesia. The uterine horns were exposed after laparotomy. Electroporation was performed using a square wave electroporator (ECM 830, BTX) and tweezer-type platinum disc electrodes (5mm-diameter, Sonidel). The electroporation settings were: 4 pulses of 40 V for 50 ms with 500 ms interval. Endotoxin-free DNA was injected using a glass pipette into one ventricle of the mouse embryos. The volume of injected DNA was adjusted depending on the experiments. Plasmids were used at the following concentrations: shRNA vectors: 0.5 μg/μl (adults) or 1 μg/μl (juveniles); GluD1, ARHGEF12, MRCKα and Ppp1r12a constructs: 1 μg/μl, except the shRNA-resistant Ppp1r12a mutant (Ppp1r12a^∗^) and GluD1-mOrange which were used 0.5 μg/μl; EGFP-GPHN: 0.3 μg/μl; Crispr knock out plasmids: 0.5 μg/μl. pCAG dsRed: 0.5 μg/μl, pCAG TagBFP: 1 μg/μl, GPHN.FingR-EGFP and PSD95.FingR-EGFP: 0.7 μg/μl. AAV injection was performed at P0 on newborn pups previously in utero electroporated at E14.5-15.5. Upon hypothermia-induced anesthesia (avoiding direct contact of the animal with the ice), pups were injected in the lateral ventricle corresponding to the electroporated side using a graduated glass pipette. The volume corresponding to 3 × 10^10^ vg (viral genome) was used. Injected pups were then rapidly warmed up and kept on a heating pad set at 37°C until complete recovery. Animals were sacrificed at the indicated age by terminal perfusion of 4% paraformaldehyde (Electron Microscopy Sciences) in PBS. Unless otherwise indicated, 100 μm coronal brain sections were obtained using a vibrating microtome (Leica VT1200S, Leica Microsystems). Sections were mounted on slides in Vectashield.

#### Immunohistochemistry for confocal microscopy

Animals at postnatal day 21 were intracardially perfused with PBS and 4% paraformaldehyde (see above). After post-fixation, dissected brains were cryoprotected in 20% sucrose at 4°C for at least 16 h and then frozen at −80°C. 20 μm coronal sections were obtained using a cryostat and further processed for immunohistochemistry. Briefly, slices were incubated in 0.1% Triton X-100 and 0.25% fish gelatin (Sigma-Aldrich) in PBS to permeabilize and block unspecific staining. Primary antibodies were incubated overnight at 4°C and secondary antibodies for 3 h at room temperature under gentle agitation. Both primary and secondary antibodies were diluted in 0.1% Triton X-100 and 0.125% fish gelatin in PBS. Coverslips were mounted on slides in Vectashield (Vector Laboratories). Mouse anti-Gephyrin (Synaptic Systems Clone 7a, 1:400) and rabbit anti-GluD1 (kind gift from L. Tricoire, IBPS, Paris, France) (Benamer et al., 2018, Hepp et al., 2015) were used as primary antibodies. All secondary antibodies Alexa- (Invitrogen) or Cyanin-conjugated (Jackson ImmunoResearch) were diluted 1:500.

#### Confocal image acquisition

Confocal images were acquired in 1024x1024 mode using Leica TCS SP8 confocal laser scanning platforms controlled by the LAF AS software and equipped with a tunable white laser and hybrid detectors (Leica Microsystems) or, for slices infected with AAVs expressing TagBFP, in 512x512 mode using an inverted microscope (Nikon Ti PSF) equipped with a CSUX1-A1 Yokogawa spinning disc and an EMCCD camera and controlled by the Metamorph software (Molecular Devices). We used the following objective lenses: 10X PlanApo, NA 0.45 (identification of electroporated neurons and low magnification images) and 100X HC-PL APO, NA 1.44 CORR CS (Leica) or 100 X HC-PlanApo, NA 1.4 (Nikon) (images of spines, gephyrin and PSD-95 clusters and association between GluD1, immunostained gephyrin and PS-D95 or Gephyrin.FingRs). Images were blindly acquired and analyzed. Z stacks of images were acquired with spacing of 150 nm.

#### Electron microscopy

Anesthetized P21 mice were intracardially perfused with 2% paraformaldehyde (PFA) and 0.1% glutaraldehyde in phosphate-buffered saline (PBS) and post-fixed overnight at 4°C in 2% PFA. Coronal sections (200 μm) were obtained using a vibratome (see above) and cryoprotected overnight in 20% glycerol and 20% sucrose under gentle agitation at 4°C. They were permeabilized with 3 freeze-thawing cycles performed by floating them on liquid nitrogen in an aluminum cup. They were then extensively rinsed in PBS, and immersed for 20 min in 50 mM ammonium chloride and for 30 min in PBS with 0.1% gelatin (PBSg). For double detection of VGAT and GluD1, GluD1 labeling was performed first. Coronal sections were incubated for 60 h at 4°C with a rabbit anti-GluD1 antibody (1:1,000 dilution, see immunohistochemistry). Sections were rinsed extensively in PBSg and then incubated 6h at room temperature with a goat anti-rabbit secondary antibody coupled with nanogold particules (Nanoprobe, 1:100). Gold particles were intensified for 5 min at 20°C with HQ silver kit (Nanoprobe) in a dark room. Gold toning (Trembleau et al., 1994) was performed on the sections. They were then rinsed extensively in PBS and PBSg. For the detection of VGAT, sections were incubated for 48 h at 4°C with anti-VGAT mouse monoclonal antibody (Synaptic Systems, 1:100), rinsed extensively in PBSg and then incubated 4 h at room temperature with a biotinylated goat anti-mouse antibody (Vector Laboratories, 1:100). Detection of the biotinylated antibody was carried by the avidin–biotin complex method (Elite Vectastain kit, Vector; and Sigma fast DAB, Sigma-Aldrich). Antigen-antibody complexes were stabilized by dipping the sections for 5 min in 1% glutaraldehyde in PBS. Sections were then post-fixed for 1 h in 2% OsO4 in PBS at 4°C in the dark, dehydrated in graded ethanol and flat-embedded in epoxy resin (Araldite, Polysciences). Embedded sections were mounted orthogonally on a resin block and cut tangentially to the pial surface. To reach cortical layers 2/3, 200 sections of 1 μm thickness of tissue were removed from the onset of layer 1 using an UC6 ultramicrotome (Leica Microsystems). Ultrathin sections (70 nm, pale yellow) were contrasted with uranyl acetate and Reynolds lead citrate. Observations were performed with a TECNAI 12 electron microscope (Philips).

#### Electrophysiology

Acute coronal brain slices (300 μm thick) were obtained from juvenile (postnatal day 16-19) Swiss mice electroporated in utero with shGluD1 in pH1SCTdT2. Whole-cell patch-clamp recordings were performed in layer 2/3 cortical pyramidal neurons of the somatosensory cortex. Briefly, after decapitation the brain was quickly removed from the skull and placed in ice-cold (4°C) ‘cutting solution’ containing (in mM): 85 NaCl, 64 sucrose, 25 glucose, 2.5 KCl, 1.2 NaH2PO4, 24 NaHCO3, 0.5 CaCl2, and 7 MgCl2, saturated with 95% O2 and 5% CO2 (pH 7.3–7.4). Slices were cut using the 7000 smz-2 tissue slicer (Campden Instrument). Slices recovered in oxygenated artificial cerebrospinal fluid (ACSF) containing (in mM): 125 NaCl, 2.5 KCl, 2 CaCl2, 1 MgCl2, 1.2 NaH2PO4, 24 NaHCO3, and 25 glucose (pH 7.4), at 35°C for 10 min and then at room temperature for at least 45 min. For electrophysiological recordings, slices were transferred to a submerged recording chamber and continuously perfused at 33–34°C with oxygenated ACSF at a rate of 4-5 ml/min. Inhibitory and excitatory miniature post-synaptic miniature currents (mIPSCs and mEPSCs, respectively) were recorded at a holding potential of −60 mV in the presence of 0.5 μM TTX. mIPSCs were isolated by adding NBQX (10 μM) and D-AP5 (50 μM) to the ACSF. mEPSCs were isolated using gabazine (10 μM). mIPSCs were recorded using an intracellular solution containing (in mM): 150 KCl, 1.5 MgCl2, 10 HEPES 10, 1 EGTA, 2 NaATP, 0.5 NaGTP (pH adjusted to ∼7.3 with KOH). mEPSCs were recorded using an intracellular solution containing (in mM): 144 K-gluconate, 7 KCl, 10 HEPES, 1 EGTA, 1.5 MgCl2, 2 NaATP, 0.5 NaGTP, (pH adjusted to ∼7.3 with KOH). Access and input resistance were monitored by applying 5 mV hyperpolarizing steps of current. All drugs were obtained from Abcam.

#### Transfection and western blotting

Transfection was performed using Jet-Prime (Polyplus Transfection) according to the manufacturer protocol. Western blotting was performed using the following primary antibodies: mouse anti-HA (HA.11 Clone 16B12 Monoclonal Antibody, Covance, 1:1,000), rabbit anti-GFP (Life Technologies, 1:2,000), rabbit anti-Myc (Cell Signaling Technology, 1:1,000), rabbit anti-GluD1 (gift from L. Tricoire, 1:1000), rabbit anti-Arhgef12 (ThermoFisher Scientific, 1:1,000), rabbit anti-Ppp1r12a (ThermoFisher Scientific, 1:1,000), rabbit anti-RFP (Rockland Immunochemicals, 1:1,000), rabbit anti-SRGAP2 (1:2,000; (Charrier et al., 2012, Fossati et al., 2016), rabbit anti-GAPDH (Synaptic Systems, 1:1,000). All HRP-conjugated secondary antibodies were used at 1:30,000 dilution (Jackson Immunoresearch). Protein visualization was performed by chemiluminescence using LumiLight western blotting (Roche) or Clarity Western ECL (Biorad) substrates and ImageQuant LAS 4000 (GE Healthcare) or Chemidoc (Biorad) imagers.

#### Cell surface biotinylation

Transfected HEK cells or primary cultures of cortical neurons at 15 or 22-23 days *in vitro* (DIV) were washed 3 times in ice-cold PBS supplemented with 0.8 mM CaCl2 and 0.5 mM MgCl2 (PBS^2+^) and then incubated for 12 min at room temperature followed by further 12 min at 4°C with 1 mg/ml Sulfo-NHS-SS-Biotin (ThermoFisher Scientific) in PBS^2+^. After rinsing in ice-cold PBS^2+^, biotin was quenched in 50 mM glycine in PBS^2+^ for 10 min. Cells were scraped in NaCl-Tris buffer supplemented with protease inhibitory cocktail (Roche) and then lysed (150 mM NaCl, 50 mM TrisHCl, 2% Triton X-100, 2 mM EDTA, 1 mM PMSF, protease inhibitor cocktail) for 1 h at 4°C. Biotinylated proteins were pulled down by incubating cell lysates with neutravidin agarose beads (ThermoFisher Scientific) for 2 h at 4°C. After extensive washes, beads were resuspended in gel loading buffer (Sigma-Aldrich) and bound proteins were eluted with boiling. Relative cell surface expression levels were analyzed by western blotting. Inputs correspond to 20% of the cell surface fraction.

#### Subcellular fractionation

Subcellular fractionation was performed from Swiss P15 mouse brains. All steps were performed at 4°C. Briefly, brains were homogenized in ice-cold HEPES-buffered sucrose (0.32 M sucrose, 4 mM HEPES pH 7.4, 5 mM EDTA, 5 mM EGTA, protease inhibitor cocktail, from Sigma) using a motor driven glass-teflon homogenizer. The homogenate was centrifuged at 3,000 g for 15 min. The resulting supernatant was centrifuged at 38,400 g for 15 min, yielding the crude synaptosomal pellet. The pellet was then subjected to hypo-osmotic shock and centrifuged at 38,400 g for 20 min. The resulting pellet was lysed for 1 h using HEPES-buffered NaCl (100 mM NaCl, 4 mM HEPES pH 7.4, 5 mM EDTA, 5 mM EGTA, protease inhibitor cocktail) supplemented with 1% CHAPS (Sigma) and centrifuged at 100,000 g for 1 h. The corresponding supernatant is referred to as synaptic fraction or synaptic membranes. Protein concentration was measured and protein samples were subjected to immunoprecipitation.

#### Immunoprecipitation

For HEK cells, 1 mg of total protein from each sample was diluted in NP-40 buffer (1% Igepal, 50mM Tris pH 7.4, 150mM NaCl, 2mM EDTA, protease inhibitor cocktail) and incubated overnight at 4°C, with either 5 μg of mouse anti-HA antibody (HA.11 Clone 16B12 Monoclonal Antibody, Covance) or 5 μg of mouse anti-Myc antibody (clone 9B11 Monoclonal Antibody, Cell Signaling Technology) and 5 μg of mouse IgG as negative control. Protein G-agarose beads (Thermo Fisher Scientific) were then added for 2 h at 4°C. After extensive washes (1% Igepal, 50mM Tris pH 7.4, 200mM NaCl, 2mM EDTA, protease inhibitor cocktail), the beads were resuspended in gel-loading buffer and bound proteins were released with boiling. Inputs correspond to 50 μg of proteins. Samples were subjected to western blot analysis. For brain samples and mass spectrometry analysis, the immunoprecipitations were performed using antibodies covalently cross-linked to protein G magnetic beads (Pierce). 36 μg of rabbit anti-GluD1 antibody, or total rabbit IgG in control condition, were incubated 1 h at room temperature and cross-linked with 20 mM DMP (dimethylpimelimidate, Pierce) in 0.2 M Sodium Borate pH 9. After 30 min, the reaction was blocked for 1 h with 0.2 M Ethanolamine pH 8. Eventual unbound antibody molecules were washed out by incubating beads for 5 min in 0.1 M glycine pH 3. The efficiency of cross-linking was checked by running samples on polyacrylamide 4%–15% gradient gels (Biorad) followed by Comassie Blue staining. 1 mg of total proteins from purified synaptic membranes were diluted in a HEPES-NaCl buffer (20 mM HEPES pH 7.4, 150 mM NaCl, 5 mM EDTA, 5 mM EGTA, protease inhibitor cocktail) supplemented with 1% CHAPS and incubated overnight at 4°C with 36 μg of rabbit anti-GluD1 antibody, or total rabbit IgG in control condition, covalently cross-linked to protein G magnetic beads. The beads were rinsed 3 times using HEPES-NaCl buffer supplemented with 0.1% CHAPS and further washed 3 times in a buffer containing 20 mM HEPES pH 7.4 and 150 mM NaCl. The samples were then subjected to mass spectrometry analysis (see below). GluD1-immunoprecipitation from brain extracts was repeated three times.

#### Proteomics

Proteins on magnetic beads were washed twice with 100 μL of 25 mM NH_4_HCO_3_ and we performed on-beads digestion with 0.2 μg of trypsine/LysC (Promega) for 1 h in 100 μL of 25 mM NH_4_HCO_3_. Samples were then loaded onto a homemade C18 StageTips for desalting (principle by stacking one 3M Empore SPE Extraction Disk Octadecyl (C18) and beads from SepPak C18 CartridgeWaters into a 200 μL micropipette tip). Peptides were eluted using 40/60 MeCN/H_2_O + 0.1% formic acid and vacuum concentrated to dryness.

Online chromatography was performed with an RSLCnano system (Ultimate 3000, Thermo Scientific) coupled online to an Orbitrap Fusion Tribrid mass spectrometer (Thermo Scientific). Peptides were trapped on a C18 column (75 μm inner diameter × 2 cm; nanoViper Acclaim PepMap 100, Thermo Scientific) with buffer A (2/98 MeCN/H_2_O in 0.1% formic acid) at a flow rate of 4.0 μL/min over 4 min. Separation was performed on a 50 cm x 75 μm C18 column (nanoViper Acclaim PepMap RSLC, 2 μm, 100Å, Thermo Scientific) regulated to a temperature of 55°C with a linear gradient of 5% to 25% buffer B (100% MeCN in 0.1% formic acid) at a flow rate of 300 nL/min over 100 min. Full-scan MS was acquired in the Orbitrap analyzer with a resolution set to 120,000 and ions from each full scan were HCD fragmented and analyzed in the linear ion trap.

### Quantification and Statistical Analysis

#### Confocal image analysis

Gephyrin and PSD-95 clusters, dendritic spines and the association of GluD1 with Gephyrin and PSD95 were quantified in the proximal part of oblique dendrites directly originating from the apical trunk using Fiji (Schindelin et al., 2012; https://fiji.sc/). Only dendrites that were largely parallel to the plane of the slice and acquired from sections of comparable rostro-caudal position were analyzed (usually no more than 1 dendrite per neuron). The density of dendritic spines and gephyrin clusters along dendrites was calculated as described (Charrier et al., 2012, Fossati et al., 2016). Gephyrin and PSD-95 clusters were quantified over a dendrite of a minimal length of 60 μm. The length of the dendritic segment was measured on the z projection. The fraction of gephyrin and PSD-95 clusters associated with GluD1 (association index) was manually determined on individual dendrites. A gephyrin or PSD-95 cluster labeled with EGFP-tagged FingRs was considered associated with GluD1 if it overlapped with a GluD1-mOrange puncta.

#### mEPSC and mIPSC analysis

Data were sampled at 10 kHz and filtered at 2 kHz. Miniature currents were analyzed over 1 min periods using pClamp 10.0 (Molecular Devices). Cells showing > 20% change in access and input resistance upon application of 5 mV hyperpolarizing steps of current were excluded from the analysis. Overlapping events were excluded from amplitude analysis. Cumulative probability graphs were obtained by taking the first 200 events within the analyzed time window of each recorded cell.

#### Proteomic analysis

For protein identification, data were searched against the *Mus musculus* (Mouse) UniProt database using Sequest HF through proteome discoverer (version 2.1). Enzyme specificity was set to trypsin and a maximum of two missed cleavage site were allowed. Oxidized methionine, N-terminal acetylation, and carbamidomethyl cysteine were set as variable modifications. Maximum allowed mass deviation was set to 10 ppm for monoisotopic precursor ions and 0.6 Da for MS/MS peaks. The resulting files were further processed using myProMS (Poullet et al., 2007) v3.6 (work in progress). FDR calculation used Percolator and was set to 1% at the peptide level for the whole study.

#### Statistics

Data are a minimum of three independent experiments. For in utero electroporations and AAV injections, data were obtained from at least three experiments or three animals from two independent litters.

For statistical analysis, we first checked the normality of the distributions using the D’Agostino-Pearson normality test. In case of normal distributions, we used unpaired Student’s t test or one-way analysis of variance followed by Tukey’s post test. Non-normal distributions were assessed using the non-parametric Mann-Whitney test or the Kruskal-Wallis test followed by the Dunn’s multiple comparison test. A test was considered significant when p < 0.05. Data represent the distribution (or the mean) of the mean value per cell in the main figures. The whiskers of the boxplots in Figure 2 are the minimal and maximal values. Statistical analyses were performed with Prism (GraphPad Software).

### Data and Code Availability

The mass spectrometry dataset generated in this study has been deposited to the ProteomeXchange Consortium via the PRIDE (Vizcaíno et al., 2016) partner repository with the dataset identifier PXD010373.

## Supplemental Information

Document S2. Table S1

Document S3. Article plus Supplemental Information
