# Supplementary material for: Trans-Synaptic Signaling through the Glutamate Receptor Delta-1 Mediates Inhibitory Synapse Formation in Cortical Pyramidal Neurons
Source: Neuron. 2019 Dec 18;104(6):1081–1094.e7. doi: 10.1016/j.neuron.2019.09.027 (PMC6926483; doi:10.1016/j.neuron.2019.09.027)
Supplement: Document S1. Figures S1–S4 [file mmc1.pdf]

Neuron, Volume *104*

Supplemental Information

Trans-Synaptic Signaling through the Glutamate Receptor Delta-1 Mediates Inhibitory Synapse Formation in Cortical Pyramidal Neurons

Matteo Fossati, Nora Assendorp, Olivier Gemin, Sabrina Colasse, Florent Dingli, Guillaume Arras, Damarys Loew, and Cécile Charrier

SUPPLEMENTAL INFORMATION

SUPPLEMENTAL DATA

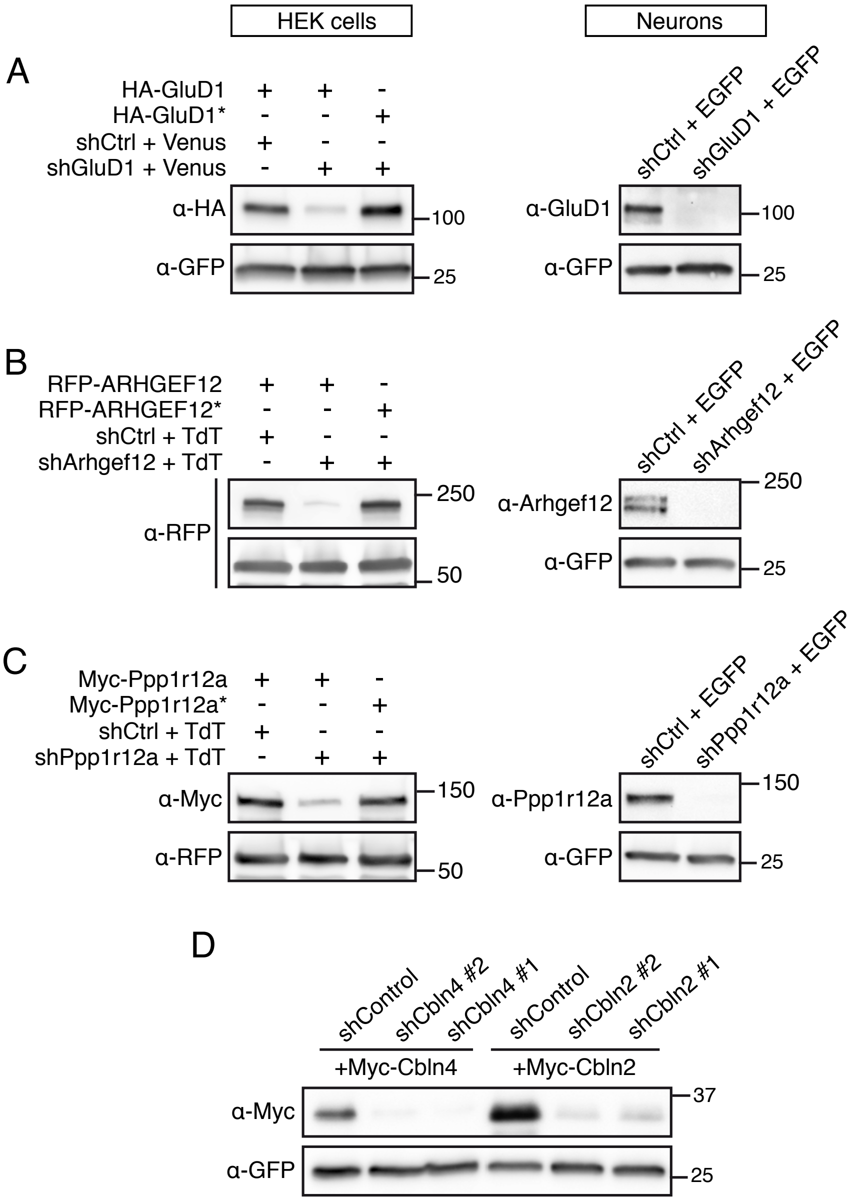

Figure S1. Validation of the shRNAs targeting *grid1*, *arhgef12*, *ppp1r12a*, *cbln2* and *cbln4* (related to Figures 1, 5 and 6).

(A-C) Validation of shRNAs targeting *grid1* (encoding GluD1) (A), *arhgef12* (B) and *ppp1r12a* (C) in HEK cells transfected with the indicated cDNAs (left panels) and in cortical neurons infected with lentiviral vectors driving the expression of shRNAs along with EGFP (right panels). HEK cells were transfected and harvested after 48 hours. Cortical neurons were infected at DIV 4 and collected at DIV 21. Cells were then lysed and subjected to western blot analysis as described in the method section. shCtrl: control shRNA; TdT: tdTomato. Constructs labeled with asterisks correspond to shRNA-resistant mutants for rescue experiments.

(D) Validation of shRNAs targeting *cbln2* and *cbln4* in HEK cells (see methods for details). shCbln4 #1 and shCbln2 #2 were selected and inserted into AAV vectors to knockdown the expression of *cbln4* and *cbln2* in mouse cortices in vivo (Figure 5).

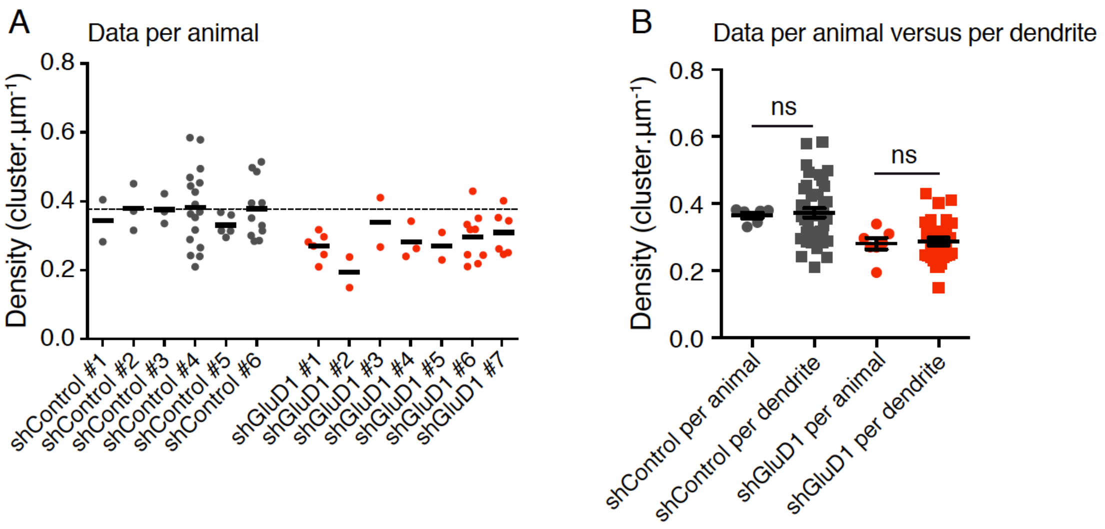

Figure S2. EGFP-gephyrin cluster analysis per animal (related to Figure 1).

(A) Plot showing the distribution of gephyrin cluster density per animal in layer 2/3 pyramidal neurons expressing a scramble shRNA (shControl) and against mouse *grid1* (shGluD1) at juvenile stage (P20-22). Each dot represents one dendrite and the bars indicate the mean value. The dotted line is the average density of gephyrin clusters in control neurons (same data as in Figure 1). #: mouse identification.

(B) Comparison of EGFP-gephyrin cluster density when the dendrites are averaged per animal or analyzed individually. Per animal analysis: $n_{shControl} = 6$, $n_{shGluD1} = 7$. Per dendrite analysis: $n_{shControl} = 41$, $n_{shGluD1} = 30$. Bars represent mean ± SEM, ns: $p > 0.05$ determined by Mann-Whitney test.

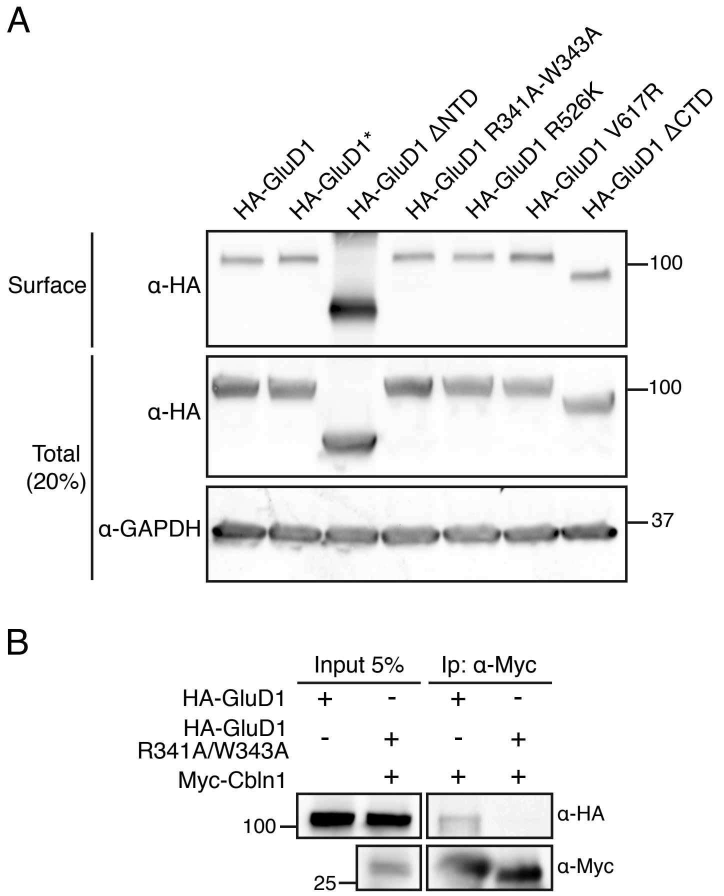

Figure S3. Characterization of GluD1 mutants (related to Figure 4).

(A) HEK cells were transfected for 24 hours with GluD1 mutants and their cell surface expression was probed with cell surface biotinylation followed by western blot. GAPDH expression level was used as a loading control. All GluD1 mutants were trafficked to the plasma membrane and their relative expression levels at the cell surface were quantified as surface/total ratio and normalized to HA-GluD1* mutant (HA-GluD1: 0.78; HA-GluD1 ΔNTD: 5.3; HA-GluD1 R341A/W343A: 1.4; HA-GluD1 R526K: 0.8; HA-GluD1 V617R: 1.7; HA-GluD1 ΔCTD: 1.6). Note that all mutants are delivered to the cell surface at least as efficiently as HA-GluD1, demonstrating that the phenotypes observed in Figure 4 cannot be ascribed to a defect in protein transport. Also, note that the overexpression of GluD1 increased the density of gephyrin clusters (Figure 1), further highlighting the importance of the NTD of GluD1 in the regulation of inhibitory synapse formation.

(B) Coimmunoprecipitation (coIP) of HA-GluD1 or HA-GluD1 R341A/W343A mutant with Myc-Cbln1 in HEK cells. Lysates of cells transfected with HA-GluD1 or HA-GluD1 R341A/W343A and lysates derived from cells overexpressing Myc-Cbln1 were mixed together and subjected to immunoprecipitation and western blot analysis (see methods for details). Note the absence of interaction between Cbln1 and the HA-GluD1 R341A/W343A mutant.

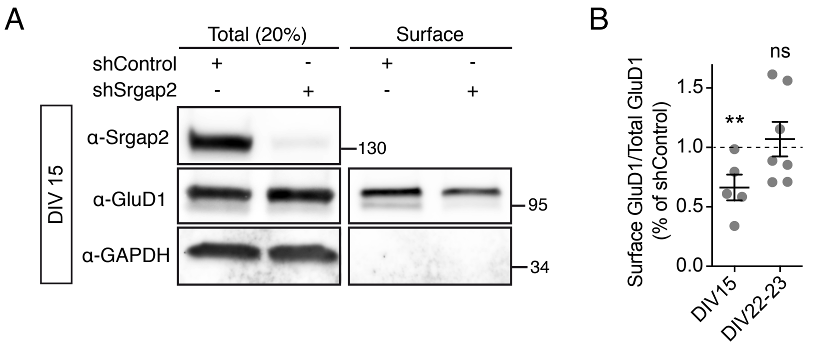

Figure S4. SRGAP2 controls the trafficking of GluD1 in cortical neurons (related to Figure 6).

(A) Cell surface expression of GluD1 using cell surface biotinylation followed by western blot in dissociated cortical neurons at 15 days in vitro (DIV). Neurons were infected at DIV4 with lentiviruses carrying either a control shRNA (shControl) or an shRNA against *Srgap2* (shSrgap2). GAPDH expression level was used as a loading control and to check the specificity of cell surface labeling.

(B) Quantification of cell surface expression levels of GluD1 in neurons expressing shSrgap2 at DIV15 and DIV22-23. Data are normalized to control neurons (dotted line). n_{DIV15} = 5 cultures, $n_{DIV22\text{-}23}$ = 7 cultures. Bars represent mean ± SEM, **: $p < 0.01$ and ns: $p > 0.05$ determined by Mann-Whitney test.

Lentiviral constructs were previously described in (Fossati et al., 2016).
